# Diagnosis and complications of renovascular hypertension in children: literature data and clinical observations

**Published:** 2009

**Authors:** Gheorghe Burnei, Anca Burnei, Dan Hodorogea, Isabela Drăghici, Ileana Georgescu, Costel Vlad, Ştefan Gavriliu

**Affiliations:** * Paediatric Surgery and Orthopaedics Clinics, “M.S. Curie” Emergency Hospital for Children, Bucharest; ** Obstetrics and Gynecology Clinics, “Elias” Emergency Hospital, Bucharest

**Keywords:** renovascular hypertension in children, diagnosis, complications

## Abstract

**Introduction**:

Renovascular hypertension in children is a very rare illness. It occurs as a result of the imbalance between hypotensive and hypertensive systems. Renal ischaemia (95% of the cases) and the shortening of hipotensive factors (5% of the cases) are responsible for the production mechanism of renovascular hypertension in children. In order to make an early diagnosis regarding the renovascular hypertension in all children suffering from renovascular illnesses, blood pressure must be taken correctly and repeatedly.

**Materials and methods**:

This paper is a case study on 19 children with renovascular hypertension, aged between 2 and 15 years old. Most cases were divided into two groups: subjects aged 4-7 years old and subjects aged 8-12 years old. Each group represents 34,2% of all cases. The diagnosis of renovascular hypertension in those 19 children was established after correctly taking the blood pressure and comparing it with the normal values for each age. Hypertension was diagnosed before knowing its cause in 8 neglected cases. The blood pressure was repeatedly taken in the other 11 children suffering from renovascular illnesses and the diagnosis of hypertension was early established when blood pressure values increased. Previously, blood pressure was normal in these 11 cases.

**Results **:

The etiopathogenical diagnosis showed parenchymal diseases in 12 cases - 63,1%. Seven patients suffered from renovascular lesions - 36,9%. Laboratory exams, radiology, imagistic exams, arteriography and scintigraphy were steps taken in order to establish the etiopathogenical diagnosis. These exams showed the next direct causes of renovascular hypertension: bilateral chronic pyelonephritis in 4 cases - 21,4%, hydronephrosis in 3 cases - 16,2%, congenital renal hypoplasia in 2 cases - 10,4% and doubled kidney in 2 cases - 10,4%. The other 8 cases presented acute glomerulonephritis, unilateral renal agenesis, horseshoe kidney, unilateral pyelonephritis, renal artery agenesis, renal trauma, renal abcess and Wilms tumor, one case of each illness - 5,2%. The major complications were: retinopathy, chronic renal failure and stroke.

**Conclusions **:

Laboratory data are just a hint in diagnosing renovascular hypertension. However, radiology, imagistic exams, arteriography and scintigraphy are compulsory in the renourinary status and etiopathogenical diagnosis.

## Introduction

The diagnosis of renovascular hypertension is established by correctly taking the blood pressure and comparing the results with normal values for each age.

Normal values of blood pressure in children according to age are showed below:

**Table:** 

Age	Normal values (mmHg)
0 - 2 years old	100 / 50
2 - 5 years old	110 / 60
5 - 10 years old	120 / 70
10 - 14 years old	130 / 80
over 15 years old	140 / 90

In order to avoid stressing the child and obtain values close to normal, blood pressure is taken after lying in bed for 15 minutes.

Blood pressure is taken three times at 10 minutes intervals after the 15 minutes repose. The value close to one of the other two values is considered the real blood pressure value.

Hypertension in adults is often idiopatic. However, hypertension in children and adolescents is a hint for a renal illness like acute glomerulonephritis, chronic glomerulonephritis, pyelonephritis, renal malformation or, rarely, coarctation of the aorta, pheocromocitoma, primary hyperaldosteronism, Cushing syndrome.

The cause of hypertension is established after gathering data from:

- Anamnesis: the presence of a renal illness or malformation.

- Clinical exam: signs of renal injury and hypertension.

- Laboratory and paraclinical exams: ecography, intravenous urography, cystoscopy, retrograde pyelography, mictional cystography, renal scintigraphy, arteriography and renal biopsy.

## Materials and methods

Among the patients of our study (**Table 1**) we have found four cases of aberant renal arteries, four cases of pyelonephritis, two cases of renopyeloureteral duplication with congenital megaureter and one case of transversal renal rupture, renal agenesis, horseshoe kidney, glomerulonephritis and Wilms tumor.

**Table 1:** The diagnoses and blood pressure values for 19 children with renal hypertension

Patient	Sex Age	Blood pressure (mm Hg)	Renal etiology	Treatment
1. P.I.	female 14 years old	160/110	Aberant renal arteries	Mixed
2. C.G.	male 14 years old	200/150	Right kidney hypoplasia. Bilateral reflux.	Surgical
3. C.M.	male 6 years old	140/90	Transversal rupture of inferior pole of the right kidney	Surgical
4. B.D.	female 10 years old	175/105	Chronic pyelonephritis	Medical
5. B.D.	male 11 years old	210/150	Chronic nephritis	Medical
6. O.L.	male 15 years old	150/90	Chronic pyelonephritis	Medical
7. G.M.	male 12 years old	140/80	Right kidney agenesis	Medical
8. D.R.	female 2 years old	110/50	Horseshoe kidney	Mixed
9. G.H.	male 6 years old	135/80	Bilateral renopyeloureteral duplication. Bilateral vesicoureteral reflux.	Surgical
10. B.T.	male 9 years old	110/70	Bilateral renopyeloureteral duplication.Right vesicoureteral reflux.	Surgical
11. C.I.	male 5 years old	120/70	Bilateral congenital megaureter	Surgical
12. D.R.	male 6 years old	140/80	Right megaureter with vesicoureteral reflux	Surgical
13. P.M.	male 4 years old	120/70	Focal glomerulonephritis	Mixed
14. R.G.	male 10 years old	175/110	Left kidney inferior polar artery	Surgical
15. T.G.	male 11 years old	140/100	Left kidney pyelonephritis	Surgical
16. B.T.	male 5 years old	140/100	Left kidney pyelonephritis	Surgical
17. P.I.	male 15 years old	170/110	Superior and inferior aberant polar arteries	Surgical
18. C.G.	female 14 years old	240/130	Right kidney hypoplasia	Surgical
19. S.M.	female 2 years old	110/70	Wilms tumor	Surgical

Among our patients, the direct causes of renal hypertension are showed in the table below:

**Table 2:** The renal diseases which caused hypertension in our 19 children

RENAL DISEASE	PERCENTAGE (%)
A. Parenchymal diseases and malformations	63,1
1. Bilateral diseases	26,3
*- glomerulonephritis*	*5,2*
*- chronic pyelonephritis*	*21,1*
2. Unilateral diseases	36,8
*- renal malformations*	*31,6*
* - renal agenesis*	*5,3*
* - congenital renal hypoplasia*	*10,5*
* - horseshoe kidney*	*5,3*
* - doubled kidney*	*10,5*
*- unilateral pyelonephritis*	*5,2*
B. Renal vascular lesions	36,9
1. Direct vascular lesions	0
2. Non-stenotic congenital vascular anomalies	5,2
*- renal artery agenesis*	*5,2*
*- aberant renal vessels*	*0*
3. Indirect vascular lesions	31,7
*- Wilms tumor*	*5,3*
*- renal trauma*	*5,3*
*- hydronephrosis*	*15,8*
*- renal abcess*	*5,3*

In our study, the parenchymal diseases predominated (12 cases of 19 – 63,1%) having only 7 cases with renovascular lesions – 36,9%. Among the parenchymal diseases seven are unilateral, six renoureteral malformations and a pyelonephritis, five are bilateral, four pyelonephritis and a glomerulonephritis (**Table 2**).Those seven cases of renovascular lesions include six indirect lesions (three hydronephrosis, one Wilms tumor, one renal abcess, one renal trauma) and one case of congenital vascular anomaly without stenosis (renal agenesis).

## Investigations

**Laboratory data**. The illnesses producing renovascular hypertension may also induce the urinary syndrome. In our study the following laboratory data were observed:

**Table:** 

Laboratory data	Cases	Percentage
Urinary infections	9	47,4%
Proteinury	4	21,1%
Leucocytury	3	15,8%
Absence of laboratory data	2	10,5%
Hematury	1	5,2%

Congenital renoureteral malformations induced many cases of urinary infection – 9 patients (47,4%). Five cases of urinary infection also presented renal lesions and only 3 patients had leucocytury - 15,8%. The proteinury - 21,1% - was moderate under 1-2 grams ‰. Studying 300 cases of idiopatic hypertension and 34 of renovascular hypertension, Bedos et al.[**1**] conclude that the presence of proteinury is worthless. In 2 cases - 10,5% - the laboratory data were missing and paraclinical exams showed right renal agenesis or horseshoe kidney, explaining the hypertension. The hematury was observed in one case - 5,2% - with dark urine and focal glomerulonephritis. The poor symtomatology made the diagnosis in horseshoe kidney case difficult. Dajani [**2**,**3**] observes three types of horseshoe kidney:

a) patients without clinical and laboratory signs;

b) cases with nausea and abdominal pain;

c) horseshoe kidney with secondary lesions (complications like infections, lithiasis, hypertension).

**Radiological and imagistical exams**

The evolution of radiological exams made the complex investigation of the entire reno-urinary tract possible.

The radiological investigation became the most important step in diagnosing urinary illnesses in children. The radiological exams used in diagnosing renovascular hypertension are: ecography, urography, cystoscopy, retrograde pyelography, mictional cystography and renal arteriography.

a) *The duplex color **Doppler ultrasonography*** is the less invasive and the less expensive screening method[**4**]. The advantages of this method include gathering data about renal artery and its intrarenal branches blood flow and the possibility of calculating the resistivity index (R.I.). This index correlates to the fibrous and atrophic lesions induced by renal artery stenosis, the main cause of renovascular hypertension in children. [**5**]

The ultrasonography is an efficient method in determining the renal size. The susceptibility of renal artery stenosis is suggested by any difference between the kidneys over one centimeter in the absence of a known renal pathology. [**4**]

The disadvantages of the method are: the prolonged examining time - over an hour, the dependence of the results on examiner’s skills and on patient’s characteristics (young child unable to cooperate with the examiner, gaseous distension of the bowels, obesity). [**6**] The sensitivity and specificity of the method as its predictive value are different from one case to another. This screening method of the renal artery stenosis is available mostly in specialized centers. [**7**]

b) *The **urography*** shows parenchymal and pyelocaliceal alterations consecutive to renal vascular anomalies or congenital malformations. We noticed the following urographic aspects among our cases:

**Table:** 

Urographic signs	Cases	Percentage
Secretion and concentration alterations	6	31,8%
Vascular print on lumbar ureter	4	21,1%
Doubled kidney	2	10,5%
Small kidney on the affected side 2 10,5%	2	10,5%
Silent kidney in urography	2	10,5%
Interrupted ureteral tract	1	5,2%
Absence of opacity in urogram	1	5,2%
Horseshoe kidney	1	5,2%
Total	19	100%

**Fig. 1:**
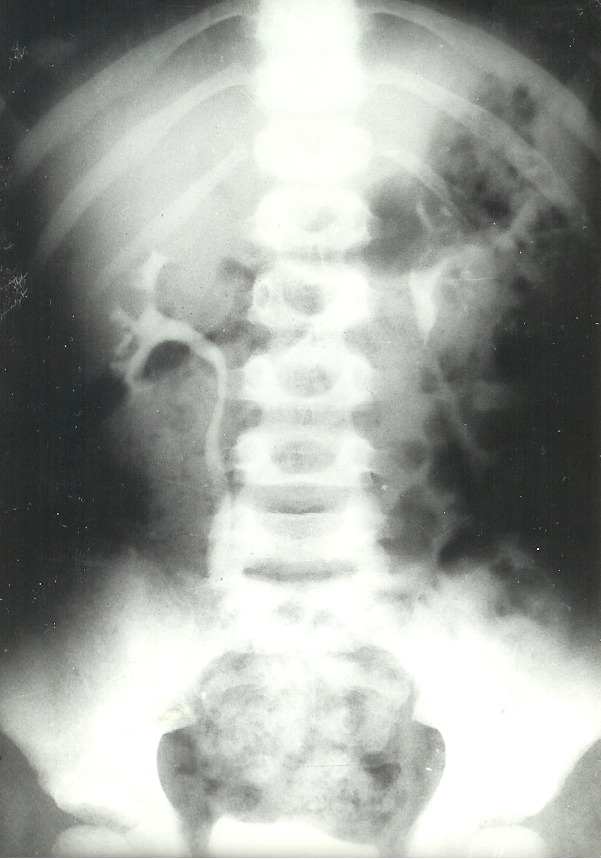
Intravenous urography. Renal asymmetry in a child with atrophic pyelonephritis of the left kidney and 140/100 mm Hg blood pressure.

**Fig. 2:**
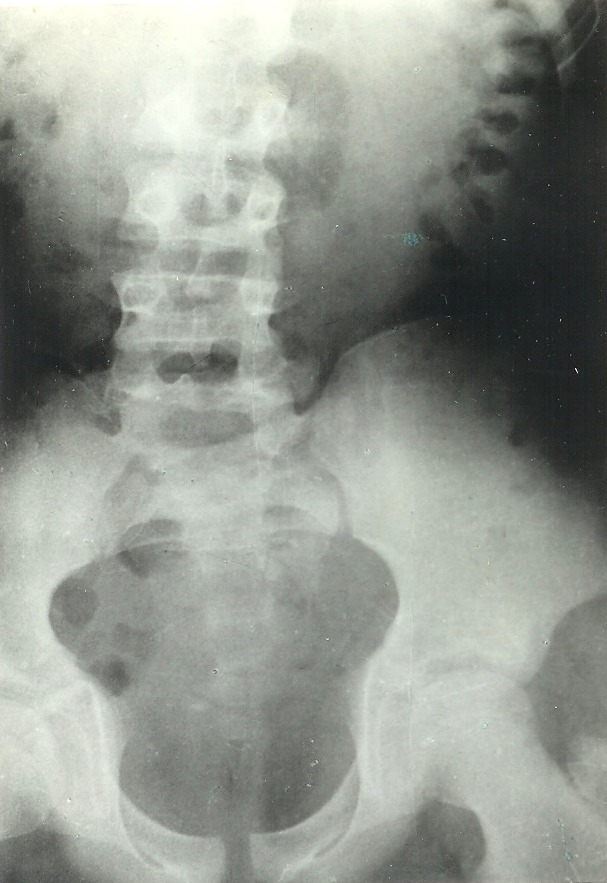
Severe bilateral secretion and concentration alterations.

Most frequent - 31,8%, the urography showed secretion and concentration alterations determined by urinary infection that involved renal parenchyma (**Fig. 1** and **2**). In 4 cases - 21,1%, the urography suggested an aberrant renal vessel due to the vascular print on lumbar ureter. The doubled kidney found in 10,5% of the cases was revealed by a bilateral doubled renal shadow on urography. All the other urographic aspects in the table counted for 5,2% of all cases. Studying 334 patients with hypertension, Bedos et al. [**8**] found other signs besides intravenous urography in 82% of the cases: abdominal murmur, albuminuria or isotopic renogram alteration.

c) *The **cystoscopy*** is performed when we suspect a vesicoureteral reflux. This exam, performed either on full or empty bladder, provides important data about the size of the ureteral orifice, the development of trigonal muscle fibers, the number of vesical wall trabecles and the laterally movement of ureteral orifices when bladder is filling. [**9**] Besides inflammatory lesions, vesical trabeculisation or a diverticle close to meatus, the cystoscopy can evaluate:

- ureteral meatus position;

- ureteral meatus morphology;

- submucous ureteral route lenght.

d) When renal function is poor, the ***retrograde pyelography*** is performed in order to observe the whole excretive tract. The retrograde pyelography was used in 2 cases of all 19 children - 10,5%.

**Fig. 3:**
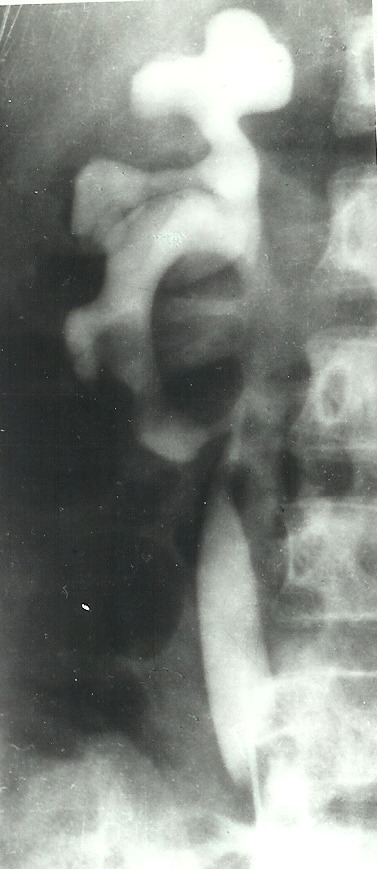
Retrograde pyelography. Right hydronephrotic dilatation due to an aberrant polar vessel.

*Observation 1 The patient C.G., aged 14, had secretion and concentration alterations that led to the impossibility of excretive tract visualisation. Retrograde pyelography was performed and we observed hydronephrosis due to an aberrant renal vessel on a right hypoplasic kidney (**Fig. 3**). The retrograde pyelography occured during the second admission when concentration and secretion alterations were evolved*.

The urography and descendent cystography suggested 12 cases of vesico-ureteral reflux. For confirmation, mictional ***cystography*** was performed using contrast into bladder and producing three pictures: after filling the bladder, mictional and postmictional. The mictional cystography is a benign radiological exam. If performed correctly, using the probes only once, it would not determine uretral trauma, or infections. The exam slightly irradiates the gonads so it is better not to use it very often. In our study, the vesicoureteral reflux was confirmed in 6 cases. Three were normal cases, 2 patients had inflammatory collum and one child had vesical residuum. We noted below the described aspects:

**Table 3:** Mictional uretrocystography in 12 children with renal hypertension.

Uretrocystographic signs	Cases	Percentage
Vesico-ureteral reflux	6	50%
Normal	3	25%
Inflammatory and spastic collum	2	16,7%
Vesical residuum	1	8,3%
TOTAL	12	100%

**Fig. 4:**
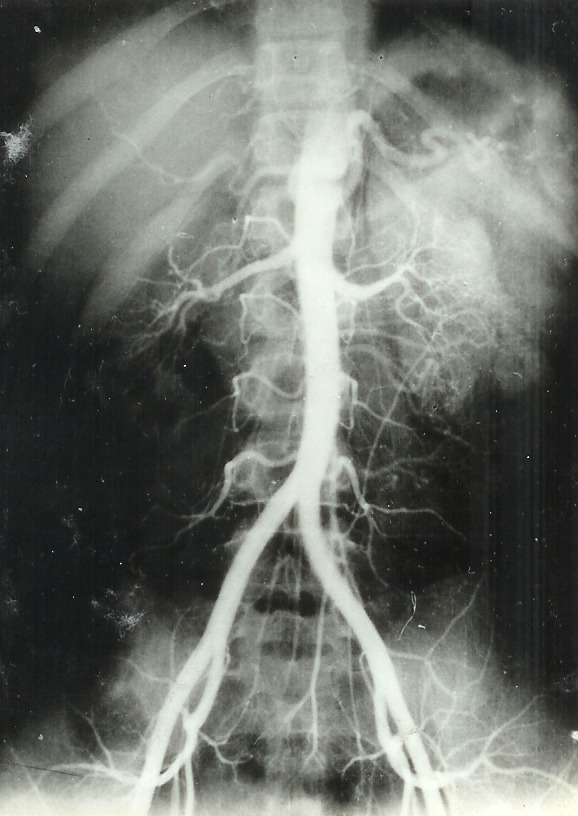
Normal arteriography in a child with chronic nephritis.

e) The ***arteriography*** is the method of choice [**10**] in diagnosing hypertension due to renal artery stenosis. This exam was performed when the urography revealed a print on the lumbar ureter - 4 cases and in other 3 cases when isotopic renogram showed renal function alterations. The arteriography did not show any sign of renal artery stenosis but only 5 cases of aberrant polar vessels. Studying data from three authors (676 cases), Pison [**11**] found renal anomalies in 26,6% of the cases. The arteriography is always to be done in hypertension due to renal illnesseses [**1**] (Bedos şi colab. 1975) because clinical data cannot orientate the diagnosis. This exam is not performed in order to confirm the diagnosis (**Fig. 4**). However, the arteriography is not used in all cases of renovascular hypertension because it can be followed by complications (renal artery thrombosis, posttrauma aneurism, fever, lumbar pain, rash).

**Isotopic nefroscintigram and angiotensin-converting enzyme inhibitor test**

Renal scintigraphy was used until the sixties. Modern tests consist in doing a series of scintigraphies: one simple scintigraphy at the begining and another scintigraphy 60 minutes after the administration of an angiotensin-converting enzyme inhibitor – captopril. The marker used in glomerular ultrafiltration is 99Tc-m-DTPA (dietil triamino-penta-acetic acid). [**12**] If renal artery stenosis is unilateral, the angiotensin-converting enzyme inhibitor administration will diminish the glomerular ultrafiltration of homolateral kidney and increase ultrafiltration of the contralateral kidney. The angiotensin-converting enzyme inhibitor test just intensifies the existent modifications on the standard nefroscintigram. [**4**]

The disadvantages of this exam are: low sensitivity in renal failure, the difficulty of interpreting the results in renal artery bilateral stenosis, the fake positive results in renal failure of any etiology. [**13**] The sensitivity of the method varies between 75% and 85%. [**14**]

Among our patients, the scintigraphy was performed in one case after the urography revealed a silent kidney. The scintigraphy showed a non-functional kidney and indicated the nephrectomy.

*Observation 2 A child aged 5, male, is admitted with a urinary infection on a renal malformation (left hypoplasic kidney). The urography showed the renal shadows asymmetry. The renal scintigraphy revealed lack of isotopic captation in the base of the right kidney. Preoperative, the uroculture was negative. Left nephrectomy is performed. Histopathological exam diagnosed scleroatrophic chronic pyelonephritis*.

## Discussions

**A. Diagnosis of hypertension in bilateral renal illnesses**

Most of the renal parenchymal illnesses can lead to hypertension. Some of them, like glomerulonephritis, renal amyloidosis, and collagenoses are bilateral, other, like chronic pyelonephritis (most of the cases) and congenital parenchymal nephropathies are unilateral. Our study includes 12 cases - 63,1% of parechymal illnesses. In order to apply the proper treatment the diagnosis was established first, weather the nephropathy was unilateral or bilateral. This way, the cases which needed to be treated surgically by nephrectomy were selected.

In order to diagnose *chronic pyelonephritis* it is necessary to reveal the renal infection and its result on kidney. Next tests are performed to show the renal infection:

1. cytobacteriologic exam– definitive 

- leucocytury over 2000/minute or over 10/mm3 urine;

- bacteriuria over 100.000 germs/ml urine.

2. Addis-Hamburger test, fractioned, provides the spot of the infection.

The following are important in order to appreciate the renal consequences of the infection:

1. instalation of hypo-, iso- or underisostenuria;

2. presence of Sternheimer-Malbin cells;

3. leucocytes cylinders;

4. renal biopsy – definitive test;

5. urography reveals pyelonephritis through: renal shadows asymmetry, silent kidney and basinetal hypotonia;

6. cystography – may show vesicoureteral reflux;

7. nephroscintigram.

*Observation 3 Child aged 14, female, is admitted with fever, polakiuria, cephalea and asteny. Laboratory exams discovered an unusual amount of leucocytes and haematies in the urinary sediment, moderately increased urea and uric acid, but the uroculture revealed over 100.000 E. coli/ml urine. The urography illustrates the pyelocaliceal nephrotic dilatation. The retrograd pyelography shows a double obstruction on lumbar ureter and arteriography, a right inferior polar artery*.

The frequency of hypertension in pyelonephritis varies from an author to another. Friedman [**15**] concludes that histological aspects of chronic pyelonephritis were found in 13,5% to 51,5% of patients with renovascular hypertension. After studying 1325 renal biopsies, Casado [**16**] found chronic pyelonephritis in 150 cases - 11,3%.

The diagnosis of glomerulonephritic hypertension is set while being based on distinctive elements for this illness: hydropigen syndrome, urinary syndrome, cardiovascular syndrome and renal failure manifestations. In our study we had one case of focal glomerulonephritis - 5,2%. Diagnosis is made on hematury, eritrocytes cylinders and moderate proteinury (0,5-1 g‰). These laboratory data frequently appear during a renal infection. The patient has no edema and the hypertension appears episodically in this illness. During the relapses, urinary alterations become permanent and lead to hypertension. There are many types of histopatologic lesions: focused and segmented, endo- mixed proliferative, intra- and exocapillary.

*Observation 4 The patient P.M., aged 4, is admitted with fever, dark urine, lack of appetite and oliguria. Laboratory exams showed: ASLO - 1250U, ESR - 110/140, urea - 1,25g‰ (after treatment – 0,26g‰), uric acid – 0,075g‰ (after treatment – 0,043g‰), urine summary – some albumin, many leucocytes grouped in conglomerates, many haematies. The blood pressure oscillated between 120/70mmHg and 110/60mmHg. Clinical aspect and the repeated acute amygdalites in antecedents suggested a focal glomerulonephritis developed on a urinary malformation: meatum stenosis with hypospadias*.

Studying 72 adults with renovascular hypertension Ursea [**17**] identifies 33 patients with glomerulonephritic hypertension: 24 chronic glomerulonephritis, 5 focal glomerulonephritis, 2 proliferative glomerulonephritis and 2 membranous glomerulonephritis. The glomerulonephritis and pyelonephritis are both medical illnesses. The frequency of these illnesses in paediatric surgery is determined by the association with renal malformations in the same children: vesico-ureteral reflux due to an improper implantation of ureters into urinary bladder, aberrant renal vessels or renopyeloureteral duplication.

**B. Diagnosis of hypertension in unilateral renal illnesses**

1) Renal malformations do not lead to hypertension in all cases. The ectopies, horseshoe kidney and doubled kidney lead to the development of hypertension in most of the cases.

*a) Congenital renal hypoplasia* was diagnosed in 2 cases - 10,5%. In the first case it was associated with a bilateral congenital megaureter and in the second case with renal artery agenesis.

*Observation 5 After multiple admissions, the patient, aged 14, is transffered from an ophtalmological clinic to establish the diagnosis. The patient is admitted with retinopathy and hypertension - 240/160mmHg. Clinical exam showed discreet renal edema. The laboratory exams excluded pyelonephritis and pheocromocitoma: urine cytobacteriologic exam was negative and vanilmandelic acid dosage in urine was normal (2,52mg‰). Renal functional exams were also normal. We thought of a renal malformation and performed a urography that showed a unique right kidney and suggested a left polar vessel, based on a tonus alteration of the left ureter, without dilatation*.

**Fig. 5:**
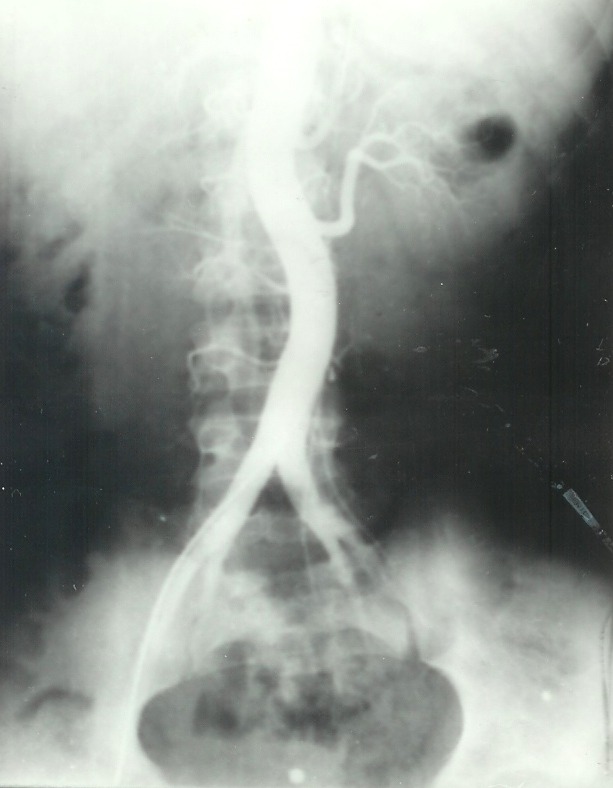
Aortography and renal arteriography. Right hypoplasic kidney

*The aortography and renal arteriography show a very small, ratatinated kidney on the right side during the nephrographic phase (**Fig. 5**). On the left, two large renal arteries vascularise a big kidney. The cystoscopy reveals increased and prolonged ureteral orifices. The cystography leads to bilateral vesicoureteral reflux as diagnosis*. 

**Fig. 6:**
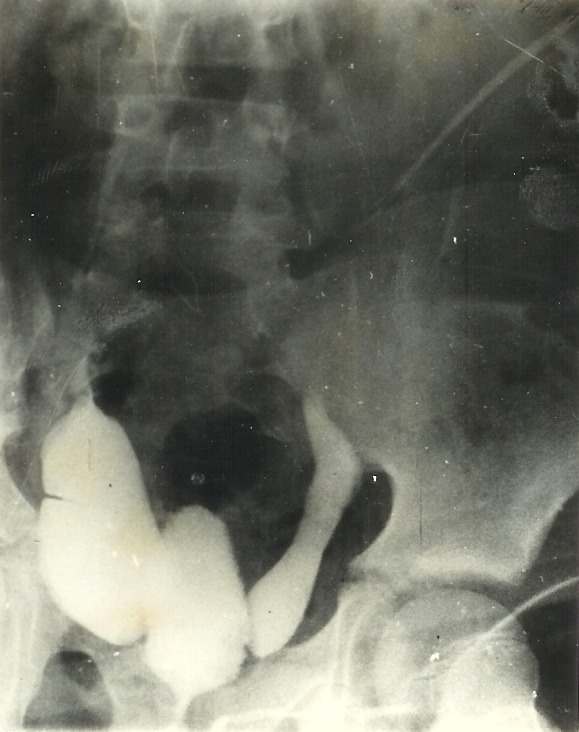
Important bilateral vesicoureteral reflux.

*Surgery is performed for hypoplasic kidney and bilateral vesico-ureteral reflux (**Fig. 6**). The volume of the right kidney is significantly diminished but its architecture is normal. We practiced right nephrectomy. The histopatological exam on the removed kidney indicated an aglomerular segmental hypoplasia. The hypertension became moderate from severe after surgery. The cytobacteriological exam revealed a renal infection after 30 days. The vesico-ureteral reflux and especially the urinary infection with reflux on unique kidney imposed the ureter reimplantation through Leadbetter – Politano method. Subsequently, the blood pressure became normal*.

*b) The doubled kidney* is different from the supernumerary kidney. This malformation consists of a big, unilateral or bilateral kidney with two basinets and distinctive vascularisation for the two segments of its construction. This kidney has a symphysis at the adjacent poles. Sometimes the borderline between the two segments is difficult to trace. The diagnosis is establised on urography that shows the volume of the kidney and its doubled basinet and ureter with ureterohydronephrosis. Our study noted two cases - 10,5% of bilateral doubled kidney with pyeloureteral bifidity. Niculescu [**18**] did not find hypertension in any of the three cases of doubled kidney studied. Tcherdokoff si Milliez [**10**,**19**,**20**,**21**] found this anomaly in one of 21 hypertensive patients and in one of 152 normotensive patients.

*c) Renal agenesis* represents the lack of any renal tissue. The excretive function is performed by the controlateral kidney if it is normal. In such cases, renal agenesis is discovered at routine investigations for local and general illness or at necropsy. In our case, the renal agenesis was associated with an ectopic cystic dilatation of the right ureter. The patient was admitted for enuresis. The urography revealed a normal left kidney and lack of opacity of the right kidney. Multiple radiotransparent images were contoured over the bladder. The renal scintigraphy showed the absence of the right kidney. The hypertension and the right ectopic ureter suggested a hypoplasic kidney although the scintigraphy showed the lack of any renal tissue on the right side. Literature data did not signalize any case of an ectopic cystic dilatated ureter on the same side with the missing kidney. The discussions over the case revealed the fact that some physicians consider it impossible for hypertension to appear in a case with an absent kidney, the contralateral kidney is healthy and a ureter malformation is present. The exploratory laparatomy elucidated the case and demonstrated in the same time that the presence of a ureteral malformation can trigger the hypertensive system even on a healthy functional kidney. This anomaly is found between 1,3 and 1,6‰ at necropsy.

*d) The horseshoe kidney* rarely determines hypertension. The diagnosis is easily established because the urography is characteristic. Nicanor Coban [**22**,**23**] described one case of horseshoe kidney accompanied by hypertension. Dajani noticed a sign that can be patognomonical in an astenic child with minimal elevated blood pressure: the hyperextension of the rahis produces a pain that ceases when the column comes to normal.

2) Unilateral chronic pyelonephritis was present in the case of a single patient - 5,2%. The diagnosis was paraclinical: Congenital hypoplasic kidney. Histopatological exam showed a sclero-atrophic pyelonephritis after nephrectomy. In this case, the hypoplasic aspect of the left kidney on urography was determined by the renal parenchyma atrophy. Vexler [**24**] considered renal hypoplasia to be a parenchymal atrophy.

**C. Diagnosis of hypertension in renovascular illnesses**

In order to diagnose direct vascular lesions some steps must be followed: clinical examination, case selection for arteriography, arteriography and the determination of stenosis functional significance. 33,6% of 104 arteriographies are found abnormal by Chamberlin [**25**], but only 4 - 3,8% of these benefit from surgical treatment. Indirect vascular lesions were present in 6 of the 19 cases, but only 3 of the 6 cases had hydronephrosis.

The laboratory exams have the privilege to diagnose hydronephrosis: plain radiography and urography. These investigations establish the diagnosis of hydronephrosis. The type of hydronephrosis, primary or secondary, is revealed by ecography, scintigraphy and arteriography. The obstructive urologic illnesses with acute manifestations may induce hypertension to one patient of ten. Studying 252 patients with obstructive uropathy, Schwartz [**26**,**27**] found an increased incidence of hypertension in acute illnesses (30% of 30 cases).

The diagnosis of Wilms tumor and renal abcess was obviously after urography.

## Complications

After studying all 19 cases we have found the following complications: retinopathy (4 cases), chronic renal failure (2 cases) and stroke (one case).

**Table:** 

Complication	Cases	Percentage
Retinopathy	4	21,1%
Chronic renal failure	2	10,5%
Stroke	1	5,2%

Most authors include retinopathy in hypertension symptomatology. We consider it a late manifestation of the renovascular hypertension in children and include it among the complications. A precocious diagnosis of renovascular hypertension followed by an adequate treatment avoids this severe complication.

The chronic renal failure is caused by progressive renal parenchyma alteration due to bilateral chronic pyelonephritis. In the end, those patients need renal transplant.

The stroke was determined by the increased blood pressure. Astasia and pyramidal syndrome occured at the hospital discharge. During subsequent admissions for medical check-ups, the neurological manifestations disappeared.

## Conclusions

Laboratory data (urinary infection, proteinury, leucocytury, hematury) are just a hint in diagnosing renovascular hypertension.

Urinary infections were observed in 9 patients -47,4%. The proteinury and leucocytury occured in 4 patients - 21,1% and respectively in 3 patients - 15,8%.

The radiological and imagistical investigation became the most important step in diagnosing urinary illnesses in children. The ultrasonography, intravenous urography, retrograde pyelography and mictional cystography revealed indirect signs of vascular malformations and showed urinary tract malformations. As urographic signs, the most frequently found were secretion and concentration alterations in 6 patients - 31,8% and vascular print on lumbar ureter in 4 patients - 21,1%. Mictional cystography confirmed vesicoureteral reflux in 50% of cases.

The arteriography is the best method to diagnose renal artery stenosis as a cause of hypertension. In our study, the arteriography did not show any renal artery stenosis but only five cases - 31,7% - of aberrant renal vessels.

The scintigraphy shows the functional remaining parenchyma in case of silent urographic kidney. This situation was noted in one case - 5,2%. 

Renovascular hypertension caused major ophtalmological complications (retinopathy - 21,1%), renal complications (chronic renal failure - 10,5%) and neurological complications (stroke - 5,2%).

## References

[1] Bedos F, Setoain J, Domenech-Torné F.M (1975). The diagnosis of renal hypertension. J Urol Nephrol (Paris).

[2] Dajani  A.M (1966). Horseshoe kidney: a review of twenty-nine cases. Br J Urol.

[3] Dajani  A.M (1983). Parapelvic cyst in a horseshoe kidney. Br J Urol.

[4] Sinescu I, Gluck G (2008). Urology.

[5] Ikee  R, Kobayashi  S, Hemmi  N (2005). Correlation between the resistive index by Doppler ultrasound and kidney function and histology. Am J Kidney Dis.

[6] Dean  M. R (2002). Renal artery stenosis: significance and treatment. J HK Coll Radiol.

[7] Krumme  B, Hollenbeck  M (2007). Doppler sonography in renal artery stenosis – does the Resistive Index predict the succes of intervention?. Nephr Dial Transpl.

[8] Duran L, Bedos F (1961). The radiology of arterial hypertension. Acta Iber Radiol Cancerol.

[9] Geavlete P (1999). vol II. Urology.

[10] Tcherdakoff  P, Milliez  P (1961). Renal arteriography: indications and contraindications in arterial hypertension. Rev Prat.

[11] Pison  C, Leveille  J, Karakand  Y, Vallieres  B (1978). Diagnostic study of nephro-urologic pathology in nuclear medicine. Union Med Can.

[12] Krumme  B, Allenberg  J. R., , Dulău-Florea  I (2005). Renovascular hypertension. Oxford Textbook of Clinical Nephrology.

[13] Pedersen E. B (2000). New tools in diagnosing renal artery stenosis. Kidney Int.

[14] Garovic  V. D, Textor S. C (2005). Renovascular hypertension and ischemic nephropathy. Circulation.

[15] Lautin E.M, Gordon P.M, Friedman  A.C, Dourmashkin L, Fromowitz  F (1979). Emphysematous pyelonephritis: optimal diagnosis and treatment. Urol Radiol.

[16] Montero Garía A, López de Novales E, Alvarez  Grande J, Casado Pérez S, Sánchez Sicilia L, Hernando  Avendaño L (1970). Major complications of percutaneous renal biopsy. Rev Clin Esp.

[17] Ursea N, Ionescu-Tîrgovişte C (1971). Renal hypertension.

[18] Niculescu I, Rosenbaum S, Andrei A, Wechsler L, Romanescu E (1960). Unusual reno-ureteral anomaly bilateral double ureter, emptying into the vagina. J Urol Medicale Chir.

[19] Milliez P, Tcherdakoff P (1961). Data obtained by certain modern methods of renal explorations (isotopic nephrograms, retrograde cystography, arteriography, cavography). J Urol Nephrol (Paris).

[20] Milliez P, Lagrue G, Tcherdakoff P, Noix M, Samarco P (1961). The new methods of investigation in renal diseases. Presse Med.

[21] Tcherdakoff P, Milliez P (1961). Incidence of congenital renoureteral anomalies in hypertensive patients. Sem Hop.

[22] Coban N, Fruchter Z (1971). Renovascular hypertension in children. Diagnosis, surgical treatment, results – National Conferrence of Urology. Cluj.

[23] Coban N, Pitea P, Popescu  Miclosan S Incidence of arterial hypertension in children with malformations of the urinary tract treated in Grigore Alexandrescu Hospital in the last 10 years – National Congress of Paediatrics, Bucharest, 29 Sept. – 2 Oct. 1965.

[24] Vexler L, Romanescu I, Marcela  Circei-Grigoriu  (1970). The small unilateral kidney and its urological implications. J Urol Nephrol (Paris).

[25] Chamberlin H.A, Hovenanian M.S (1953). Aneurysm of accessory renal artery. J Urol.

[26] Garcia-Peña B.M, Keller M.S, Schwartz D.S, Korsvik H.E, Weiss R.M (1997). The ultrasonographic differentiation of obstructive versus nonobstructive hydronephrosis in children: a multivariate scoring system. J Urol.

[27] Dell'Aria JC, Petrilli R, Schwartz E (1988). Acute occlusion of the left renal artery manifested by hypertensive crisis. J Emerg Med.

